# The fair decision-making of children and adolescents with high-functioning autism spectrum disorder from the perspective of dual-process theories

**DOI:** 10.1186/s12888-020-02562-8

**Published:** 2020-04-06

**Authors:** Peiying Jin, Yao Wang, Yun Li, Yunhua Xiao, Chunyan Li, Nana Qiu, Jiao Weng, Hui Fang, Xiaoyan Ke

**Affiliations:** Nanjing Brain Hospital Affiliated to Nanjing Medical University, 264 Guangzhou Road, Gulou District,Nanjing, Jiangsu Province, 210009 China

**Keywords:** High-functioning autism spectrum disorder, Fair decision-making, Theory of mind, Executive functioning, Dual-process theories

## Abstract

**Background:**

Fairness has received much attention in our society. At present, the findings regarding fair decision-making in high-functioning autism spectrum disorder (HF-ASD) are inconsistent. Previous studies have shown that the fair decision-making of typically developing children is influenced by theory of mind (ToM) and executive functioning (EF). As those with HF-ASD have defects in both domains, this study aims to explore the differences in fair decision-making between children and adolescents with HF-ASD and those with typical development (TD).

**Methods:**

We used a simple ultimatum game (UG) to explore 31 children and adolescents with HF-ASD and 38 children and adolescents with TD. T tests and chi-square tests were used to compare group differences, and Pearson correlation analysis and stepwise regression analysis were used to analyse the mechanisms influencing the two groups’ unfair acceptance rates.

**Results:**

The results show that children with HF-ASD are more likely to accept unfair offers, but for adolescents, the difference is not significant. Regression analysis showed that the interaction between the behavior regulation index (BRI) and age could negatively predict the unfair acceptance rate of children and adolescents with HF-ASD. Working memory and ToM can negatively predict the unfair acceptance rate of those with TD.

**Conclusion:**

This study concluded that the development of fair decision-making by children and adolescents with HF-ASD falls far behind that of those with TD. Intuition processes play a dominant role in the fair decision-making processes of children and adolescents with HF-ASD, and we believe that comorbidity, age, experience and emotional management are important factors influencing the fair decision-making of individuals with HF-ASD.

## Background

Fairness is the quality of treating people equally or in a way that is reasonable. A good society should enable people to experience stable and lasting cooperation, and only fairness can realize this. In our daily lives, we often pay attention to fairness and are willing to punish unfair behaviour. Therefore, fairness, as a core component of moral society, has received much attention from psychologists and economists [1, 2]. Autism spectrum disorder (ASD) is a severe neurodevelopmental disorder that begins in early childhood and is characterized by impairment in social communication and interaction and repetitive behaviours or interests [3]. ASD comprises heterogeneous neurodevelopmental disorders with mild to severe clinical symptoms, and many ASDs are accompanied by intellectual diseases [3]. In the clinic, individuals with ASD whose IQs are equivalent to or higher than those of normally developing individuals are classified as having high-functioning autism spectrum disorder (HF-ASD). In society, compared with individuals with other ASDs, individuals with HF-ASD may be more likely to acquire the ability to study and live independently [4]. However, it is difficult for those with HF-ASD to establish friendships with others and cooperate with each other because of their deficits in social communication and interaction [5]. Those with HF-ASD are at great risk of being bullied by typically developing peers in school or other places due to a lack of social skills, gradually leading to subsequent emotional and psychological problems and violent behaviours [6, 7]. Some researchers have claimed that this risk may result from their misunderstanding of other people’s intentions and their perception of unfair treatment, which results in HF-ASD children being less tolerant of unfair treatment [7–9]. Kate Anne Woodcock et al. used an ultimatum game (UG) to study the fair decision-making of HF-ASD children between 11 and 17 years old [10]. In UG experiments, participants are given a fixed amount of funds and are assigned to a proposer or responder role. The proposer offers an allocation of funds, and if the responder accepts, the funds are dispersed according to the proposer’s allocation. If not, both parties receive an amount of 0. The results showed that there was no significant difference between those with HF-ASD and those with typical development (TD) when in the role of responder [10]. Other researchers indicated that children with HF-ASD between 6~16 years old were more likely to accept unfair distribution, and they claimed that this higher acceptance rate might be related to defects in theory of mind (ToM), which is the ability to understand and predict others’ feelings and behaviours [11, 12]. At present, research on fair decision-making in individuals with HF-ASD is primarily focused on ToM, but there are also studies showing that individuals with HF-ASD have difficulty changing strategies in gambling games, possibly due to defects in executive functioning (EF), which is a set of cognitive processes that include inhibition, shifting, monitoring, planning/organizing, and working memory that could be measured by the Behavior Rating Inventory of Executive Function (BRIEF) [11, 13–15]. However, Susan Faja et al. found that individuals with HF-ASD did not significantly differ from those with TD in social decision-making and flexibility of goal orientation [16]. Some researchers have claimed that fair decision-making not only involves ToM but also is influenced by outcomes of decision-making, that is, the rational cognitive part of fair decision-making [17]. Research on the neural mechanism of TD also shows that fairness-related decisions are regulated not only by ToM but also by a series of cognitive processes [18]. Some researchers use dual-process theories, which separate Type 1 intuitive processes from Type 2 reasoning processes, to explain the fair decision-making of those with TD [19–21]. The intuitive process is independent and does not require working memory. It is a fast, automatic process that enables the processing of large amounts of information simultaneously [19]. Some studies classify emotions as intuitive processes, while reasoning processes depend on working memory and are capable of representing reality [18]. Self-regulation, which is the ability to overcome impulses and control behaviour, can temporarily prevent the results of intuitive processing from being directly output and then reasoning processes will work, and dual-process theories hold that the interaction of cognition and intuitive response can alter the fair decision-making of those with TD [18, 22]. In addition, some researchers have used cognitive tasks (such as syllogistic reasoning or cognitive reflection tasks, CRT) to study the theory of dual processing in ASD populations and concluded that ASD populations rely more heavily on inference processing than on intuitive processing [23–25].

Compared with TD, HF-ASD leads to defects in the ability to infer others’ minds [26], which prevents individuals with HF-ASD from making appropriate social decisions that require measuring the interests of themselves and others [17]. Researchers have also found that HF-ASD impairs EF [27]. Some studies have found that EF is related to ToM in HF-ASD populations, and some researchers have claimed that EF is a prerequisite of ToM [28–31]. Therefore, we assume that EF and ToM may simultaneously affect the fair decision-making of those with HF-ASD and that the dual processing mechanism of fair decision-making in HF-ASD populations is different from that in TD populations. To our knowledge, there are only two studies on these two aspects of fair decision-making in individuals with HF-ASD. One is Wang yao et al., who discuss the influence mechanism of fair decision-making in individuals with HF-ASD from the perspective of ‘brain types’ [12]. The study found that the unbalanced development of HF-ASD ‘brain types’, that is, the imbalance between systemizing and empathy, causes individuals with HF-ASD to have greater tolerance for unfair distribution [12]. The other is Kate Anne Woodcock et al., who used the UG and found that individuals with HF-ASD are more influenced by ToM when acting as the proposer in fair decision-making games but are more influenced by EF when acting as the responder [10]. Ultimately, there are different conclusions regarding the fair decision-making of individuals with HF-ASD, and the roles of ToM and EF in the fair decision-making of individuals with HF-ASD have not been adequately taken into consideration. In contrast to the study by Kate Anne Woodcock, we recruited children and adolescents ranging from 6~16 years of age. In addition, our study aims not only to explore the differences in fair decision-making between children and adolescents with HF-ASD and those with TD but also to focus on the association between two aspects of cognition (ToM and EF) and the behaviour of two participant groups in a UG. Finally, we discuss the possible psychological mechanism behind these differences from the perspective of dual-process theories.

## Methods

### Participants

We recruited thirty-one HF-ASD participants (4 females) from outpatient clinics at the Children’s Mental Health Research Center of Nanjing Medical University Affiliated Brain Hospital and 38 TD participants (5 females) from the community.

The inclusion criteria for the HF-ASD group were as follows: [1] met the diagnostic criteria for ASD according to the Diagnostic and Statistical Manual of Mental Disorders-fifth edition (DSM-5) [2]; met the autism scoring standards of the Autism Diagnostic Interview-Revised (ADI-R) and Autism Diagnostic Observation Schedule (ADOS) (with cut-off) [3]; was 6–16 years old, with a Wechsler Intelligence Scale for Children—third edition (WISC-III) full-scale intelligence quotient (FIQ) greater than 80 [4]; received parental permission to participate in the study; and [5] was right handed.

The exclusion criteria for the HF-ASD group were as follows: [1] having a history of head trauma [2]; having a neurological or mental disorder; and [3] using neurological or psychiatric drugs.

The TD group was recruited from the general population, including community and school. The inclusion criteria for the TD group were as follows: [1] having TD (including physical, cognitive, social and sensory skill according to the parent report), with an age, sex and IQ that matched those of the HF-ASD group [2]; receiving parental permission to participate in the study; and [3] being right handed.

The exclusion criteria for the TD group were as follows: [1] having a history of head trauma [2]; having a neurological or mental disorder; and [3] using neurological or psychiatric drugs.

The study was approved by the Medical Ethics Committee of the Nanjing Medical University Affiliated Brain Hospital (KY043), and all participants signed informed consent forms.

### Materials and analysis

#### Assessment

##### Behavior rating inventory of executive function (BRIEF) [32]

Some researchers hold the opinion that the EF tests have lower ecological validity than EF ratings and may be unrelated to daily EF [33]. The BRIEF is a widely used behavioural questionnaire for parents of school-age children to assess everyday EF. It is designed for a broad range of children between 5 and 18 years of age. This scale contains 86 items, all of which are three-level scoring items. It is divided into two subindexes: the behavior regulation index (BRI) and the metacognition index (MI). The BRI includes inhibition, shift and emotional control, which is related to self-regulation. The MI includes five factors: initiative, working memory, plan/organization, organization of materials and monitoring. BRI and MI scores form the global executive composite (GEC), which represents the overall level of EF deficit. The higher the score is, the more serious the deficit.

##### Griffith empathy measure—parent report (GEM-PR) [34]

The GEM-PR is a scale of empathy for children and adolescents that is completed by parents according to the actual situation of their children. There are 23 items, all of which are scored on a 9-point scale. The higher the total score is, the greater the empathy. The total table is divided into two dimensions: cognitive empathy (GEM-C), similar to ToM, is the ability to understand emotions from the perspective of others, and affective empathy (GEM-A) involves experiences observing the emotional states of others.

##### Ultimatum game

In recent years, researchers have often used games, including the ultimatum game (UG), dictator game (DG) and prisoner’s dilemma (PD), to study fair decision-making. The UG designed by Güth Schmittberger and Schwarze is one of the most commonly used of these games [35]. In UG experiments, participants are given a fixed amount of funds and are assigned to a proposer or responder role. The proposer proposes an allocation of funds, and if the responder accepts, the funds will be dispersed according to the proposer’s allocation. If not, both parties receive an amount of 0. In our pre-experiment, it was difficult for most HF-ASD participants to remain completely focused in the paradigm. To ensure completion, a simple version of the UG was used. In the test, the subjects were assigned to only the responder role and provided with 9 different allocations. Computers presented ultra-fair (80%), fair (50%) or unfair (20%) proposals about how to divide 10 yuan, 20 yuan and 30 yuan. Each allocation was presented twice for a total of 18 rounds. Presented by E-prime 1.0, the test procedure is as follows.

Step 1: The participants entered the laboratory with the experimenter for a while to familiarize with the experimental environment.

Step 2: The operation mode is explained to the participants before the experiment, and they are then shown the instructions on a computer screen. The participants then completed a practice test. The practice allocation scheme differs from the actual allocation scheme.

Step 3: The participants’ familiarity with the task is tested through questioning, and then the experiment begins. The instructions are again displayed on the screen, and the “space bar” is pressed to start the experiment. The preparation time for each round was 2 s. For each round, the computer screen displays the proposer’s allocation for 6 s. Then, the participants are asked to respond by pressing the “accept” or “reject” button. The decision process does not exceed 6 s. Finally, the results are displayed. For example, if the participant accepts, he or she may receive 2 yuan while the proposer receives 8 yuan. If the participant rejects an offer, both the responder and proposer receive 0 yuan.

Step 4: After the game, the participant is rewarded with candy or other gifts.

#### Statistical analysis

SPSS 23.0 software was used for statistical analysis. First, independent sample T tests and chi-square tests were used to compare the differences in sex, age and IQ between the two groups. Then, chi-square tests were used to compare the acceptance rates of three different allocations between the two groups and the unfair acceptance rates of the two groups by age. Pearson correlation analysis was used to explore the influencing factors of the two groups’ unfair acceptance rates, and then, stepwise regression analysis was used to analyse the influencing mechanism of the two groups’ unfair acceptance rates.

## Results

The HF-ASD group included a total of 27 males and 4 females. The average age of this group was 9.07 ± 2.69 years, and the participants’ average IQ was 106.10 ± 17.76. The TD group included a total of 33 males and 5 females. The average age of this group was 9.72 ± 2.76 years, and the participants’ average IQ was 117.68 ± 11.32. There were no significant differences in sex, age, IQ, GEM-A or GEM between the two groups, while there were significant differences in BRIEF and GEM-C scores (*P* < 0.05) (see Table 1 for results).

**Table 1:** Demographic and clinical characteristics of ASD and TD participants

	HF-ASD (*M ± SD*)	TD (*M ± SD*)	*t/*χ^*2*^	*SE*
Sex			0.00	
Male	27	33		
Female	4	5		
Age	9.07 ± 2.69 range 6–15	9.72 ± 2.76 range 6–16	− 0.99	0.66
IQ	106.10 ± 17.76 range 80–143	117.68 ± 11.32 range 92–140	49.06	
**BRIEF**				
Inhibit	60.16 ± 10.42 range 38–76	47.85 ± 9.96 range 36–78	5.03^***^	2.45
Shift	58.87 ± 8.80 range 47–77	49.9 ± 8.58 range 36–76	4.27^***^	2.09
Emotional control	56.42 ± 10.78 range 42–76	45.82 ± 9.10 range 35–80	4.46^***^	2.38
BRI	59.65 ± 9.30 range 45–78	47.05 ± 9.88 range 36–80	5.44^***^	2.31
Initiate	60.07 ± 8.79 range 38–77	48.13 ± 7.46 range 35–66	6.14^***^	1.94
Working memory	62.97 ± 9.10 range 40–82	48.59 ± 7.34 range 38–66	7.32^***^	1.96
Plan/Organize	64.42 ± 10.06 range 45–89	52.26 ± 7.52 range 37–67	5.79^***^	2.10
Organization of materials	54.81 ± 8.28 range 39–72	49.00 ± 8.01 range 33–63	2.97^**^	1.96
Monitor	65.00 ± 9.01 range 50–82	52.26 ± 8.71 range 33–72	5.96^***^	2.14
MI	63.29 ± 8.54 range 44–85	49.97 ± 7.31 range 37–66	7.02^***^	1.90
GEC	63.00 ± 7.65 range 46–79	49.08 ± 7.91 range 36–69	7.43^***^	1.88
**GEM-PR**				
GEM-C	−0.05 ± 0.89 range − 1.50-1.67	1.16 ± 1.30 range − 1.00-3.67	−4.42^***^	0.27
GEM-A	1.16 ± 0.90 range − 1.67-3.22	0.91 ± 1.01 range − 1.44-3.33	1.07	0.23
GEM	0.69 ± 0.65 range − 0.83-2.13	0.99 ± 0.74 range − 0.74-2.96	−1.73	0.17

*BRIEF* Behavior Rating Inventory of Executive Function; *BRI* Behavioral Regulation Index; *MI* Metacognition Index; *GEC* Global Executive Composite; *GEM-PR* Griffith Empathy Measure—Parent Report; *GEM-C* Cognitive Empathy; *GEM-A* Affective Empathy; ^**^indicates *p* < 0.05, ^***^ indicates *p* < 0.001

### Acceptance rates of the two groups for different allocations

The UG results show that there was no significant difference between the two groups in the acceptance rate of ultra-fair offers (*χ*^*2*^ = 3.48, *P* = 0.06) and fair offers (*χ*^*2*^ = 0.02, *P* = 0.89). However, the acceptance rate of unfair allocations (*χ*^*2*^ = 36.40, *P* = 0.00) was significantly different, and the HF-ASD group accepted unfair allocations at a higher rate (see Table 2 for results).

**Table 2:** Acceptance rates of the HF-ASD and TD groups for different allocations

	HF-ASD(*M ± SD*)	TD (*M ± SD*)	*χ*^*2*^
Ultra-fair	86.56 ± 23.34 range 0.00–100.00	83.76 ± 30.23 range 0.00–100.00	3.48
fair	85.48 ± 25.73 range 16.67–100.00	88.46 ± 26.26 range 0.00–100.00	0.02
unfair	46.77 ± 37.86 range 0.00–100.00	22.22 ± 31.14 range 0.00–100.00	36.40^***^

Ultra-fair represents 80% of the stake; fair represents 50% of the stake; unfair represents 20% of the stake. ^**^indicates *p* < 0.05, ^***^ indicates *p* < 0.001

**Fig. 1:**
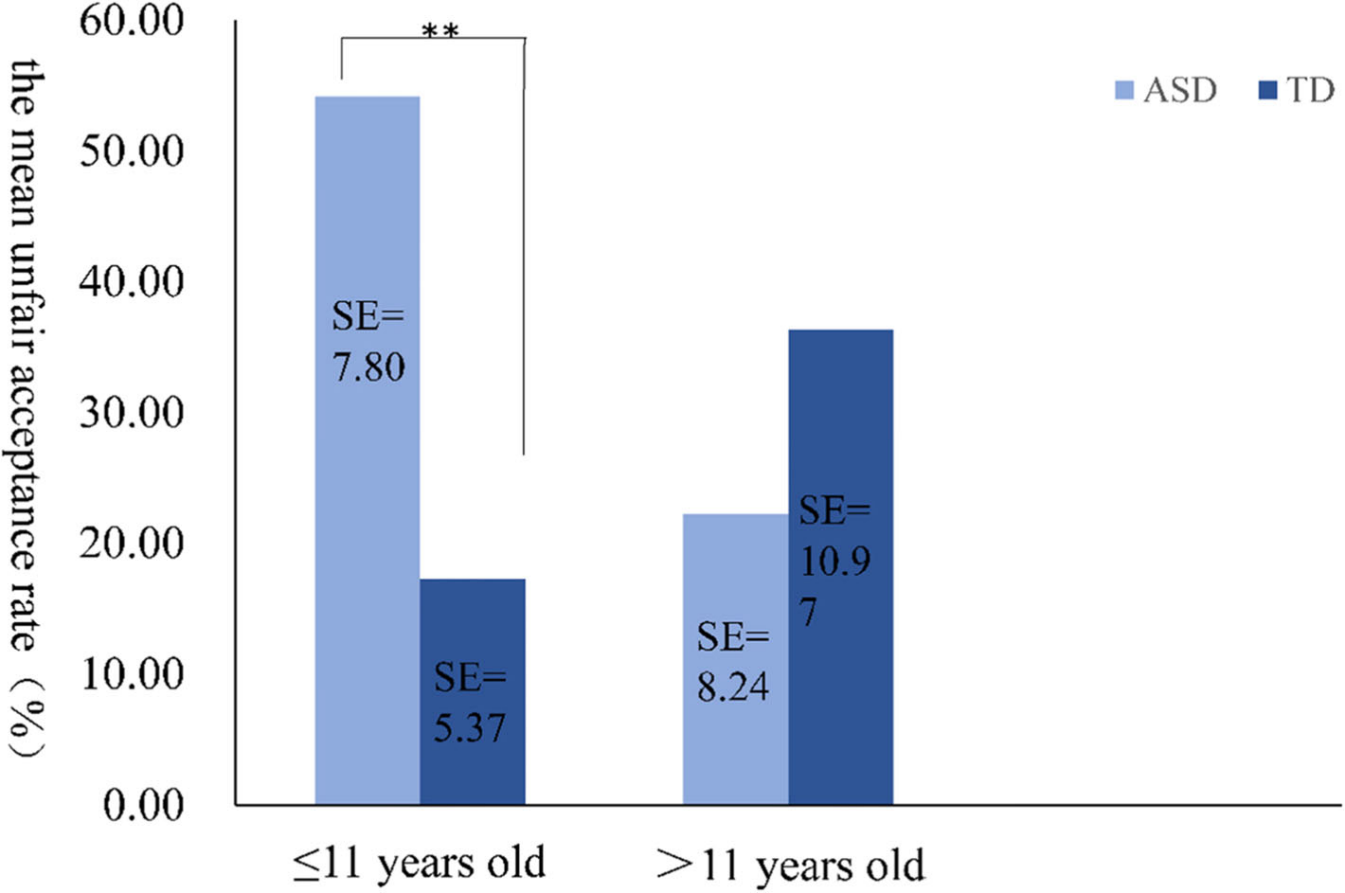
Unfair acceptance rates at different ages in both groups. ** *P* < 0.05

**Fig. 2:**
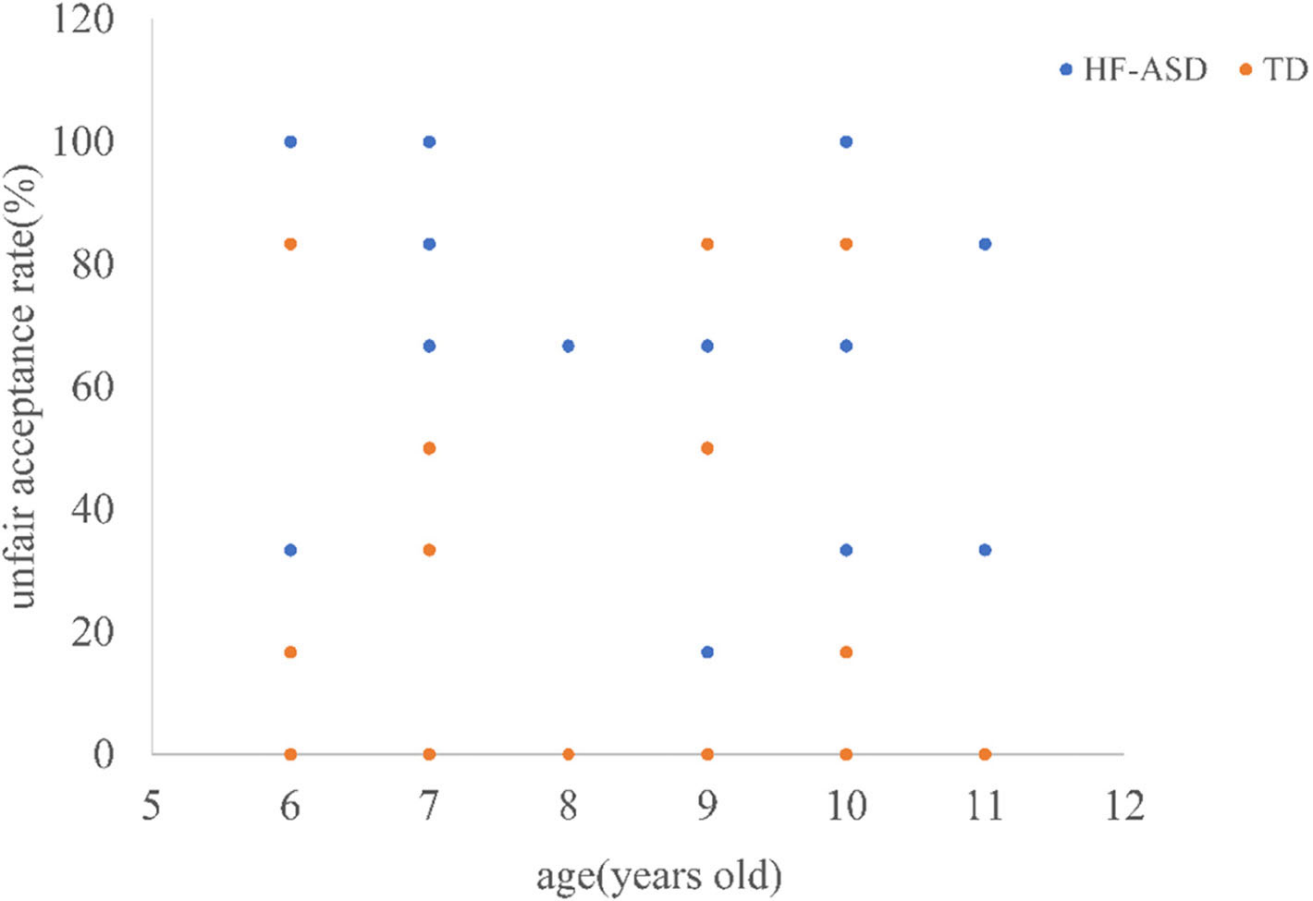
Unfair acceptance rates under 12 years old in both group

Subsequently, we explored the relationship between unfair acceptance and age in the HF-ASD (*r* = − 0.31, *P* = 0.09) and TD (*r* = 0.23, *P* = 0.17) groups, and the results showed no significant relationships. Then, we divided the HF-ASD group and TD group into childhood (≤11 years old) and adolescent (> 11 years old) groups to compare their unfair acceptance rates (Fig. 1). The unfair acceptance rate of the childhood HF-ASD group (*M* = 52.67, *SD* = 38.99) was higher than that of the adolescent HF-ASD group (*M* = 22.22, *SD* = 20.18), but the difference was not significant. Comparing the unfair acceptance rate of the HF-ASD group with that of the TD group, it was found that the unfair acceptance rate of the childhood ASD group was significantly higher than that of the childhood TD group (*χ*^*2*^ = 19.30, *P* = 0.00), but the difference was not significant for the adolescent groups (*χ*^*2*^ = 3.13, *P* = 0.97). The scattergram of age versus unfair acceptance rate of children under 12 can be seen in Fig. 2.

### Relationship between the unfair acceptance rates of the two groups and related factors

Pearson correlations found that the unfair acceptance rate of the HF-ASD group was significantly negatively correlated with BRI score (*r* = − 0.36, *P* = 0.049), while that of the TD group was significantly negatively correlated with GEM-C score (*r* = − 0.36, *P* = 0.03) (see Table 3 for results).

**Table 3:** Relationship between the unfair acceptance rates of the two groups and related factors

	HF-ASD		TD	
	*r*	*P*	*r*	*P*
Age	−0.31	0.09	0.23	0.17
IQ	−0.28	0.13	0.22	0.19
Inhibit	−0.22	0.23	0.10	0.54
Shift	−0.34	0.06	−0.03	0.84
Emotional control	−0.30	0.10	−0.08	0.64
BRI	−0.36	0.049^**^	0.01	0.94
Initiate	−0.15	0.42	−0.00	0.98
Working memory	−0.07	0.72	−0.21	0.22
Plan/Organize	0.10	0.69	−0.12	0.49
Organization of materials	−0.11	0.54	0.00	1.00
Monitor	0.02	0.92	0.12	0.467
MI	−0.03	0.86	−0.05	0.77
GEC	−0.19	0.30	0.01	0.97
GEM-C	0.05	0.80	−0.36	0.03^**^
GEM-A	−0.07	0.70	0.02	0.90
GEM	−0.02	0.92	−0.17	0.31

*BRIEF* Behavior Rating Inventory of Executive Function; *BRI* Behavioral Regulation Index; *MI* Metacognition Index; *GEC* Global Executive Composite; *GEM-PR* Griffith Empathy Measure—Parent Report; *GEM-C* Cognitive Empathy; *GEM-A* Affective Empathy; ^**^indicates *p* < 0.05, ^***^ indicates *p* < 0.001

### Stepwise regression analysis of two groups

#### Stepwise regression analysis of the ASD group

Pearson correlations found that the BRI of the ASD group was moderately correlated with total EF (GEC) (*r* = 0.69, *P* = 0.00) and age (*r* = 0.37, *P* = 0.02). Therefore, BRI, BRI*GEC and BRI*age were all included in the stepwise regression analysis. The results show that the interaction between BRI score and age in the ASD group is a negative predictor of unfair acceptance rate, with an explanation of 14.5% (see Table 4 for results).

**Table 4:** Stepwise regression analysis of the ASD and TD groups

		*B*	*SE*	*β*	*T*
**ASD**					
Included variable	Constants	84.25	18.07		4.66^***^
	BRI*age	−0.68	0.03	−0.38	−2.22^**^
Excluded variable	BRI			−0.17	−0.70
	BRI*GEC			−0.13	−0.58
**TD**					
Included variable	Constants	33.94	6.38		5.32^***^
	GEM-C*working memory	−2.11	0.08	−0.40	−2.60^**^
Excluded variable	GEM-C			0.72	0.95
	GEM-C*monitor			1.33	1.65
	GEM-C*MI			1.59	1.34
	GEM-C*IQ			0.40	0.71
	GEM-C*GEM			0.02	0.06

*BRIEF* Behavior Rating Inventory of Executive Function; *BRI* Behavioral Regulation Index; *MI* Metacognition Index; *GEC* Global Executive Composite; *GEM-PR* Griffith Empathy Measure—Parent Report; *GEM-C* Cognitive Empathy; *GEM-A* Affective Empathy; ^**^indicates *p* < 0.05, ^***^ indicates *p* < 0.001

#### Stepwise regression analysis of the TD group

Pearson correlations revealed that the GEM-C score in the TD group had a medium-low correlation with IQ (*r* = 0.35, *P* = 0.03), working memory (*r* = − 0.40, *P* = 0.04), monitoring (*r* = − 0.39, *P* = 0.02), MI score (*r* = − 0.35, *P* = 0.03) and GEM score (*r* = 0.54, *P* = 0.00); thus, GEM-C, GEM-C*IQ, GEM-C* working memory, GEM-C*monitor, GEM-C*MI and GEM-C*GEM were included in the stepwise regression model. The interaction between GEM-C and working memory was found to be a negative predictor of the unfair acceptance rates of the TD group, with a total explanation of 15.8% (see Table 4 for results).

## Discussion

This study focuses on the fair decision-making of individuals with HF-ASD and IQ- and age-matched individuals with TD in a UG. First, we explore the difference between HF-ASD and TD in fair decision-making. Second, we address the association of ToM and EF in HF-ASD and TD. Finally, we discuss the possible psychological mechanism of HF-ASD in fair decision-making, which may be different from that in TD.

### The fair decision-making difference between HF-ASD and TD

The HF-ASD group accepted unfair offers at a higher rate than the TD group, while there was no significant difference between the acceptance rates for ultra-fair offers and fair offers, which was consistent with previous findings [11, 36]. Researchers have found that typically developing children and adolescents usually reject unfair offers and, as a third party, tend to punish unfair individuals [37, 38]. Moreover, some researchers believe that human beings show a preference for fairness when they are 12 months old and can already make decisions according to each other’s distribution intentions and distribution results when they are 4 years old [39, 40]. Therefore, the results of this study suggest that the development of a sense of fairness among individuals with HF-ASD lags behind that of typically developing individuals of the same age.

Previous studies have claimed that fair decision-making in children varies with age [11, 41]. In this study, we found no relationship between unfair decision-making and age in either group. According to developmental psychology, Jean Piaget suggests that the concrete operational stage (7~12 years) is critical for social cognition development. In this study, most participants were in this age range, so it is difficult to account for a tendency towards unfair acceptance. In addition, although fairness can be well developed during childhood, younger children (9 and 12 years) and older adolescents (15 and 18 years) show differences in fair decision-making [42]. Accordingly, the HF-ASD group and TD group were stratified by age. The results show that individuals with HF-ASD are more inclined to accept unfair distributions in childhood but not in adolescence, possibly due to the small sample size in this study. The unfair acceptance rates of the two HF-ASD age groups were compared with those of the TD age groups. The unfair acceptance rates of the childhood HF-ASD group were significantly higher than those of the childhood TD group, but no significant difference was found between the two adolescent groups, possibly because children showing TD through the age of 6 have developed a sense of fairness, while children with ASD slowly develop a sense of fairness when they are teenagers.

### The association between unfair acceptance and ToM and EF in the two groups

In the general population, ToM, as the basis for cooperation, is often considered to participate in fairness-related behaviours [43, 44]. Accordingly, we found that the unfair acceptance rates of children and adolescents with TD were related to GEM, that is, ToM. Individuals with ASD are often considered to lack the ability to understand the intentions of others. In previous studies, fairness-related behaviours were shown to be related to ToM development defects [45], but no consistent conclusion was found in our study. At the same time, we found that the unfair acceptance rates of children and adolescents with HF-ASD are related to BRIEF scores, which is consistent with previous results [10, 11, 13, 14]. Further regression analysis shows that both GEM and working memory have an impact on the unfair acceptance rate of individuals with TD, while the interaction between the BRIEF scores of individuals with HF-ASD and age can negatively predict unfair acceptance rates.

### The possible psychological mechanism of HF-ASD in fair decision-making

**Fig. 3:**
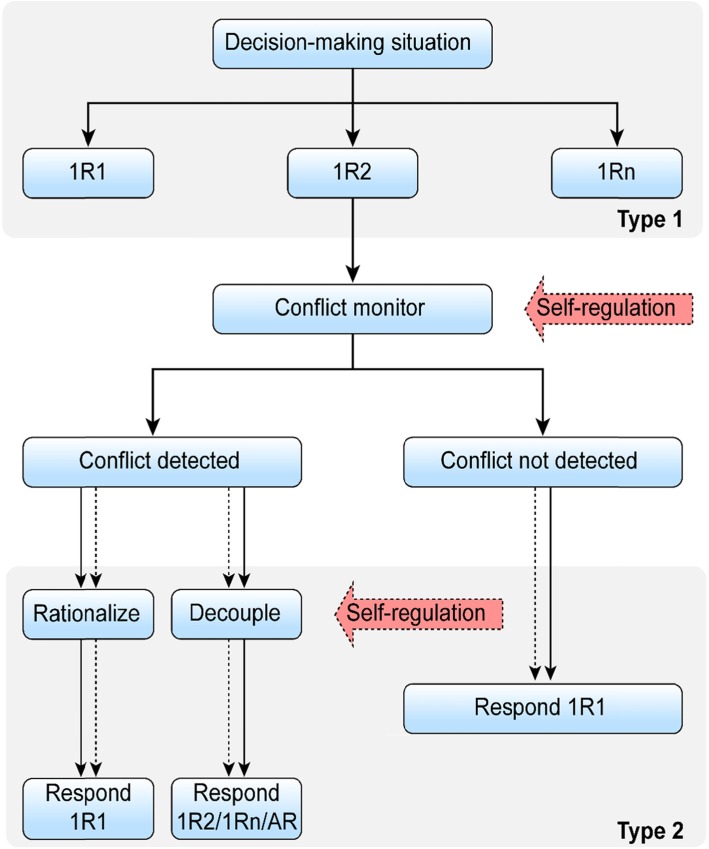
Dual-process theories of individuals with TD and individuals with HF-ASD. 1R1 is the most salient and fluent possible response, 1Rn is the other. possible intuitive reaction, and AR refers to an alternative reaction. The. dashed line represents weakened processes in individuals with ASD.

According to the dual-process theories of fair decision-making, intuitive processes are fast, nonlogical processes independent of working memory [46]. Other researchers consider emotion-related factors, such as negative emotions generated by unfair distributions, to be a result of respondents’ intuitive processes [47]. Reasoning is a slow process that depends on working memory and operates on information from the specific situation. In line with Pennycook et al. [21, 48], we believe that the fair decision-making of individuals with TD is affected by both intuitive processes and reasoning processes (see Fig. 3). However, compared with individuals with TD, those with HF-ASD have defects in ToM and EF. Some researchers claim that individuals with HF-ASD may avoid social stimulation due to these deficits, so they cannot follow the daily norms of social cooperation and cannot learn the concepts of fair and unfair from the social environment [36]. Our study found that the interaction between the BRIEF scores of individuals with HF-ASD and age can negatively predict unfair acceptance rates. Therefore, we can conclude that the dual processing of individuals with HF-ASD differs from that of individuals with TD (see Fig. 3). Self-regulation plays a role in inhibiting intuitive processes and activating the reasoning process in dual processing. Eliran Halali et al. have shown that self-regulatory depletion, which results from inhibiting related tasks before UG experiments, leads to an increase in the rejection rate of unfair distribution [49]. BRIEF scores represent the degree of effective self-regulation through effective inhibition to change cognition and then regulate emotion and behaviour. In individuals with HF-ASD, the BRI reveals deficits. Therefore, we believe that intuitive processing plays a leading role in the fair decision-making of individuals with HF-ASD. In addition, individuals with HF-ASD may be more “selfish” due to defects in ToM, which makes them seem as if they are living in their own world. They consider only their own interests and losses and will not experience negative emotions in response to receiving unfair offers. Acceptance may be intuitive to these individuals, hence their higher rates of accepting unfair offers. In this research, we did not exclude HF-ASD patients who also suffered from attention deficit hyperactivity disorder (ADHD). In our opinion, individuals with ASD comorbid with ADHD are more likely forced to undergo a series of negative social experiences regarding unfairness in social activities. In later life, negative emotions accompany similar situations. Meanwhile, behaviour management weakens with age, which leads to a failure of emotional control. In addition, the intuitive process occupies the dominant position in individuals with HF-ASD + ADHD, and experience is just a part of the intuitive process. Therefore, a selfish nature conflicts with experience, and individuals with HF-ASD + ADHD are unable to adjust and adapt, thus leading to negative emotions. Finally, individuals with HF-ASD + ADHD are unable to adjust negative emotions caused by experiences and conflicts due to poor behaviour management skills, so they are immersed in their own emotional world and experience a stronger emotional response to unfairness. Therefore, with age, their reactions to unfair events strengthen. In our sample, the dual processing mode of the HF-ASD population was dominated by intuitive processing, and this finding conflicts with previous research results on the dual processing mode of HF-ASD populations. First, the task of our study differs from those of previous studies. We use a simple gambling game that focuses on gain and loss, while previous studies have used more complex reasoning tasks (for example, if five machines need 5 min to make five widgets, how long will it take 100 machines to make 100 widgets?). Second, previous studies have focused on teens and adults over the age of 16, not children and adolescents under 16. Finally, individuals with ADHD were not strictly excluded from the HF-ASD group, so further studies are needed.

## Limitations and future direction

Our study found no significant relationship between fair decision-making and age, but a difference between HF-ASD and TD in childhood was found, possibly due to the limited age range and sample size in this study. Studies with larger age ranges and larger samples may be more promising. Fair decision-making is a complicated social behaviour. Similarly, the results of this study show that EF and ToM cannot fully predict fair decision-making in children and adolescents with TD or HF-ASD. In addition to the factors involved in the two processes studied here, reality representation capabilities may impact the rates of accepting unfair distributions. Therefore, future research may need to analyse these factors.

## Conclusion

This paper finds that the development of fair decision-making in individuals with HF-ASD lags behind that of individuals with TD, and individuals with HF-ASD are more likely to accept unfair offers. The study also finds that the interaction between BRI score and age affected the fair decision-making of children and adolescents in the HF-ASD group, which is negatively correlated with unfair acceptance. Therefore, this paper proposes that the influencing mechanisms behind the fair decision-making of individuals with HF-ASD and TD may differ. The fair decision-making of individuals with HF-ASD may be dominated by intuitive processing, and we believe that comorbidity, age, experience and emotional management are important factors influencing the fair decision-making of individuals with HF-ASD. Variations in stimulation or the environment can affect the social cognition of individuals with HF-ASD.

## Data Availability

The datasets used and/or analysed during the current study are available from the corresponding author on reasonable request.

## Abbreviations

HF-ASD: High-functioning autism spectrum disorder:
ToM: Theory of mind
EF: Executive functioning
TD: Typical development
mini-UG: Mini ultimatum game
BRI: Behavior regulation index
CRT: Cognitive reflection tasks
DSM-5: Diagnostic and statistical manual of mental disorders-fifth edition
ADI-R: Autism diagnostic interview-revised
ADOS: Autism diagnostic observation schedule
WISC-III: Wechsler intelligence scale for children—third edition
FIQ: Full-scale intelligence quotient
BRIEF: Behavior rating inventory of executive function
MI: Metacognition index
GEC: Global executive composite
GEM-PR: Griffith empathy measure—parent report
GEM-C: Cognitive empathy
GEM-A: Affective empathy
DG: Dictator game
PD: Prisoner’s dilemma
ADHD: Attention deficit hyperactivity disorder
